# Editorial: Role of Epigenetic Modifications on Diet-Induced Metabolic Diseases

**DOI:** 10.3389/fgene.2020.00825

**Published:** 2020-09-15

**Authors:** Manlio Vinciguerra, Andrea Masotti, Anna Alisi

**Affiliations:** ^1^International Clinical Research Center, St. Anne's University Hospital, Brno, Czechia; ^2^Division of Medicine, Institute for Liver and Digestive Health, University College London, London, United Kingdom; ^3^Research Laboratories, Bambino Gesu' Children Hospital-IRCCS, Rome, Italy; ^4^Research Unit of Molecular Genetics of Complex Phenotypes, Bambino Gesu' Children Hospital-IRCCS, Rome, Italy

**Keywords:** diet, metabolism, epigenetic modification, programming, liver diseases

The prevalence of diet-induced metabolic diseases, including insulin resistance, type 2 diabetes (T2D), hypertension, cardiovascular disease, non-alcoholic fatty liver disease (NAFLD) and metabolic syndrome, is rising rapidly worldwide, with obesity representing a major cause of these diseases (Han and Lean, 2016). Moreover, obesity enhances the likelihood of developing numerous blood-borne and solid cancers, thus undoubtedly increasing morbidity and mortality in an unprecedented manner (Islami et al., 2019).

The pathogenesis of metabolic diseases that are triggered by excessive caloric intake remains to be fully elucidated, although researchers have recently highlighted that organ and systemic damage associated with these pathologies might be the result of a complex network of interactions between genetics and environment, potentially orchestrated by epigenetic mechanisms (Sorrenti et al., 2017).

The eukaryotic epigenome is believed to interact with environmental stimuli through alterations of several chromatin features, and by differential silencing or activation of gene expression. Cells exhibit three major interconnected systems that are able to turn on/off genes: (1) DNA methylation, (2) histone modifications, and (3) incorporation of histone variants into the chromatin and non-coding RNAs (including microRNAs) (Jiménez-Chillarón et al., 2012). Whether dietary habits or specific nutrients during prenatal and postnatal life may trigger specific epigenetic mechanisms, which in turn are involved in the development of disease phenotypes, has not yet been fully established.

A deeper understanding of the mutual influence between dietary patterns and epigenetic modifications could have important diagnostic and therapeutic implications.

In this Research Topic, we present a series of both review articles and original research manuscripts that illuminate the role and effects of epigenetic modifications occurring in diet-induced metabolic diseases.

Epidemiological evidence has revealed that health and diseases in later life are also linked to fetal origins and early life factors, such as maternal health status and diet (Vinciguerra and Cordero Sanchez, 2020). Indeed, adverse events during prenatal life may result in permanent changes in the physiology and metabolism of the offspring, which in turn lead to programming and an increased risk of a variety of metabolic diseases.

In our Research Topic, Deodati et al. provide evidence of the epigenetic regulation (mainly DNA methylation and microRNAs) of the developmental programming of metabolism that may be related to the insurgence of cardiometabolic disease. Some external environmental factors (i.e., air pollution) coupled with a wrong diet might increase the risk of cardiovascular diseases and metabolic disorders. Barchitta et al., in their original research, evaluate the effect of particulate matter (PM) exposure and adherence to a Mediterranean Diet (MD) on the methylation of interspersed nucleotide elements 1 (LINE-1). Interestingly, this surrogate marker of global genomic DNA methylation is associated with cardiovascular disease and cancer. The authors found an inverse association between the adherence to MD and exposure to PM10 and LINE-1 methylation levels. Beside pollution, specific genotoxic insults may also affect diet and induce epigenetic modifications that may have consequences on the onset of multifactorial diseases. ADP-ribosylation is an important post-translational protein modification that regulates diverse biological processes, controlled by dedicated transferases and hydrolases. Gene copy number variants of mono-ADP-Ribosylhydrolase *MACROD2* are associated with early onset obesity and insulin resistance (Pettersson et al., 2017). However, the physiopathological relevance of these findings remain elusive. In their original research, Lo Re et al., demonstrate that newborn *MACROD2*-knock-out mice actually display no differences in irradiation-induced lethality and DNA damage, high fat-diet induced obesity, or insulin and glucose/insulin intolerance, when compared to wild type littermates. The latter study underlies the importance of *in vivo* testing, using suitable animal models, to confirm epidemiologic and *in vitro* studies.

In addition to environmental factors and diet, the complex network of interactions established between the host, its microbes, and their products within the human intestine (i.e., the gut microbiota) may influence epigenetic mechanisms in eukaryotes and induce different obesity-related diseases. In a review article, Sharma et al. discuss the potential pathophysiological effects of the gut microbiome and epigenetic crosstalk on obesity and type 1/type 2 diabetes.

DNA methylation patterns in genomic and intergenic regions might also be a contributing factor in T2D, thus suggesting that epigenetic marks could represent potential biomarkers of disease. Importantly, Willmer et al., reviewed the most recent lines of evidence about the potential use of DNA methylation patterns obtained from human blood samples as semi-invasive candidate biomarkers for T2D prevention and treatment. This tool could be particularly relevant for the evaluation of the beneficial effects of some natural and/pharmacological anti-diabetic agents (Wen et al.) by monitoring epigenetic modifications from readily available body fluids, such as blood or urine.

However, an outstanding open question remains: are epigenetic modifications causative or simply associated with the cardiometabolic risk of T2D? Greco et al. attempts to reply to this question by providing an overview of the epigenetic role and the effects of some specific nutrients and other chemicals in the regulation of metabolic chronic disorders. In any case, it appears clear that dietary habits during pregnancy may influence offspring health at birth and later in life. Accordingly, in a piece of original research, Bianchi et al. demonstrate that maternal intake of N-3 polyunsaturated fatty acids, profiled at pregnancy, impacts on the offspring's DNA methylation status analyzed by cord blood mononuclear cells. In particular, the authors show that differentially methylated genes belonged prevalently to cell signaling pathways and metabolic processes, and identified four genes (i.e., *MSTN, IFNA13, ATP8B3*, and *GABBR2*) as potential determinants of the infant's metabolic programming.

Lipid imbalance, elicited also by a threonine-deficient diet in animal models, may also increase the risk of NAFLD. Threonine and S-adenosyl-methionine metabolism can influence histone methylation. In a piece of original research, Jiang et al. showed that a threonine-deficient diet in ducks caused NAFLD by triggering a differential expression of genes that regulate triglyceride metabolism in the liver. Among the epigenetic mechanisms involved in NAFLD, there is not enough attention on histone modifications and their dynamic changes. Barbaro et al. commented on a recent original study that demonstrates that the histone demethylase plant homeodomain finger 2 protects the liver from NAFLD progression.

The understanding of how epigenomic regulation might affect individual risk of developing obesity-related comorbidities represents a major challenge, especially in the study of genes that are involved in disease phenotypes (i.e., genes that are involved in feeding and related to endocannabinoid and the opioid systems). In this respect, Pucci et al. showed that a high-fat-diet-induced epigenetic modulation of type 1 cannabinoid receptor gene and mu opioid receptor gene expression provides a new robust hypothalamic biomarker for the early development of obesity.

Finally, as we recalled before, obesity increases death rates of different types of cancers, including NAFLD-related hepatocellular carcinoma (HCC). It is widely accepted that in NAFLD-related HCC pathogenesis multiple interactions between genetics, epigenetics, and diet play a key role. In this respect, Dreval et al. showed that NAFLD-related HCC in a genetic animal model is characterized by the progressive accumulation of alterations in DNA methylation and altered gene expression patterns, and highlighted that the progressive changes in *TUBB2B* expression/methylation might be a contributor to hepatocarcinogenesis. Other authors reviewed genes and epigenetic mechanisms that are linked to HCC development (Farcas et al.; Krupenko and Horita).

In conclusion, this Research Topic was aimed at providing up-to-date evidence of the link between diet, environmental factors, epigenetic modifications, and the onset or progression of several obesity-related diseases. However, we would like to highlight here that epigenetic marks, especially those detectable in the circulation by non-invasive high-throughput approaches, might become efficient and effective predictive biomarkers of the stratification and progression of several chronic diseases that represent a costly healthcare endeavor.

## Author Contributions

All authors listed have made a substantial, direct and intellectual contribution to the work, and approved it for publication.

## Conflict of Interest

The authors declare that the research was conducted in the absence of any commercial or financial relationships that could be construed as a potential conflict of interest.
